# Hormone Therapy and Biological Aging in Postmenopausal Women

**DOI:** 10.1001/jamanetworkopen.2024.30839

**Published:** 2024-08-29

**Authors:** Yufan Liu, Chenglong Li

**Affiliations:** 1 Capital Medical University, Beijing, China; 2 National Institute of Health Data Science at Peking University, Beijing, China; 3 Institute of Medical Technology, Health Science Center of Peking University, Beijing, China

## Abstract

**Importance:**

Menopause is associated with biological aging, and hormone therapy (HT) is associated with health outcomes in postmenopausal women.

**Objective:**

To evaluate the association between HT use and discrepancies between chronological and biological age in postmenopausal women as well as the potential modifying role of socioeconomic status (SES).

**Design, Setting, and Participants:**

This population-based, retrospective cohort study included postmenopausal women registered in the UK Biobank. A baseline survey on HT use and biological aging biomarkers was conducted from March 2006 to October 2010. Data analyses were conducted in December 2023.

**Exposures:**

Information regarding HT use, the age at starting HT, and HT duration was collected via a touchscreen questionnaire. SES was evaluated by education, family income, occupation, and the Townsend Deprivation Index.

**Main Outcomes and Measures:**

Biological aging discrepancy was evaluated using validated phenotypic age, which was calculated using chronological age and 9 biomarkers measured at baseline. All-cause and cause-specific mortality were also assessed.

**Results:**

Among the 117 763 postmenopausal women (mean [SD] age, 60.2 [5.4] years), 47 461 (40.3%) ever used HT. The mean phenotypic age was 52.1 (7.9) years. Ever use of HT was associated with a smaller biological aging discrepancy than never use of HT (β, −0.17 years; 95% CI, −0.23 to −0.10 years). This smaller aging discrepancy was more evident in those who started HT at age 55 years or older (β, −0.32 years; 95% CI, −0.48 to −0.15 years) and in those who used HT for 4 to 8 years (β, −0.25 years; 95% CI, −0.35 to −0.15 years). The association between HT and a smaller aging discrepancy was more evident in women with low SES, with a significant interaction observed for education (higher education: β, −0.08 years [95% CI, −0.17 to 0.01]; other education: β, −0.23 [95% CI, −0.32 to −0.14] years; *P* for interaction = .02). Phenotypic aging discrepancy mediated 12.7% (95% CI, 6.3% to 23.9%) of the association between HT and all-cause mortality and cause-specific mortality.

**Conclusions and Relevance:**

In this study, postmenopausal women with historical HT use were biologically younger than those not receiving HT, with a more evident association observed in those with low SES. The biological aging discrepancy mediated the association between HT and decreased mortality. Promoting HT in postmenopausal women could be important for healthy aging.

## Key Points

**Question:**

Are there associations between hormone therapy (HT), socioeconomic status (SES), and discrepancies between chronological and biological age among postmenopausal women, and can this explain the association between HT and risk of mortality?

**Findings:**

In this cohort study involving 117 763 postmenopausal women, HT use was significantly associated with a smaller aging discrepancy in postmenopausal women with different SESs, with a more evident association observed in women with low SES. The association between HT and a smaller aging discrepancy mediated the association between HT and decreased mortality risk.

**Meaning:**

These findings support HT use in postmenopausal women to promote healthy aging and address related health inequalities, but further investigations are warranted to confirm the clinical benefits.

## Teaser

This cohort study evaluates whether hormone therapy use is associated with discrepancies in chronological and biological age among postmenopausal women, considering socioeconomic status as a potential modifying factor.

## Introduction

Attention to the aging process and contributing factors is increasing because of the expanding aging population worldwide. Several alternative measures have been developed to measure the aging process.^1^ Among these established measures, biological age assessed using phenotypic age is widely used.^1^ The phenotypic age is derived using a machine learning approach, capable of capturing morbidity and mortality risks across diverse populations, independent of chronological age.^2^ Previous studies have investigated the discrepancy between phenotypic and chronological age with several exposures, such as lifestyle and childhood adversity.^3^

Among the factors involved in women’s health, hormone therapy (HT) has been the focus of much attention. Menopause is associated with the loss of estrogen production by the ovaries.^4^ In addition, there is an association between menopause and the biological aging process, indicating the urgent need to address accelerated aging in postmenopausal women.^5,6^ Exogenous systemic estrogen is recommended for managing menopausal vasomotor symptoms. However, concerns have been raised regarding the health effects of HT. The Women’s Health Initiative Hormone Trials showed that in postmenopausal women, HT increased the risks of stroke and probable dementia^7,8^ while it did not reduce the incidence of coronary heart disease.^7^ In contrast, observational data from the Nurses’ Health Study showed a potentially protective role of HT against major coronary events.^9^ A review of historical evidence suggested potential reasons for the observed inconsistency between studies,^10^ such as healthy user bias inherited by observational studies and the heterogeneous timing of initiating HT, the latter of which was supported by an emulated target trial.^11^

Recently, 2 large-scale, observational studies found that HT use was associated with an elevated incidence of dementia.^12,13^ Determining the health effects of HT is important for current practice because of the large number of women receiving HT. Moreover, because the discrepancy between phenotypic and chronological age is superior to other aging measures (including telomere length) in predicting adverse outcomes,^14^ examining its association with HT use could offer meaningful insight into promoting health and preventing disease. The heterogeneous effects of HT have been explained with the timing hypothesis, partially supported by the known effects of estrogen on cardiovascular risk factors.^15^ Previous studies have found associations between the timing of initiating HT and several outcomes, including dementia and brain structure.^16,17^ However, no study has examined the timing of HT with biological aging. Therefore, this study aimed to investigate the associations between HT use, including the timing of initiating HT; phenotypic age discrepancy in postmenopausal women; and the potential modifying role of socioeconomic status (SES).

## Methods

### Study Population

The UK Biobank is a large-scale cohort of more than 500 000 men and women aged 40 to 69 years, established to support the investigation of risk factors for major diseases of middle and old age. Further details regarding the design and the survey content can be found elsewhere.^18,19^ This cohort study was approved by the North West Multi-center Research Ethics Committee, and all participants provided written informed consent before enrollment. A baseline survey on HT use and biological aging biomarkers was conducted from March 2006 to October 2010. This study followed the Strengthening the Reporting of Observational Studies in Epidemiology (STROBE) reporting guideline.

Among the original 273 301 female participants available for inclusion, we excluded 107 932 participants without menopause, 19 138 without assessment of HT usage, 62 with dementia at baseline, 512 without assessment of tobacco and nicotine exposure, and 27 894 without complete information for calculating phenotypic age. A total of 117 763 participants were included (eFigure 1 in Supplement 1).

### Assessment of HT

The information regarding HT use in the UK Biobank was obtained via a touchscreen questionnaire. The participants were initially asked “Have you ever used hormone therapy (HT)?” with potential responses including “Yes,” “No,” “Do not know,” and “Prefer not to answer.” Individuals who responded “Do not know” or “Prefer not to answer” were coded as missing and thus excluded from the analysis. Individuals who responded “Yes” were further asked 2 questions: (1) “How old were you when you first used HT?” and (2) “How old were you when you last used HT?” If individuals responded an age younger than 16 years or older than their current age at the interview, the values were then coded as missing and excluded from the analysis. If individuals answered an age younger than 35 years or older than 65 years, requests were then made to confirm their answers. Regarding the age when HT was last used, some individuals responded, “Still taking HT,” which was also coded as missing and excluded from the analysis. However, in our sensitivity analysis, we additionally retained individuals currently using HT and reanalyzed associations between HT and biological aging discrepancy by treating current HT users as a single category. Consequently, we extracted available information regarding whether HT was used, the age of starting HT, and the duration of HT use (age that HT was last used minus age of first using HT), as previously described.^12^ The duration of using HT for current HT users was calculated as the interval between age at the baseline survey and age of starting HT.

### Assessment of SES

We initially considered SES indicators of total household income before tax, education attainment, and occupation in the UK Biobank.^20,21^ We did not include health insurance because the National Health Service was established to provide a comprehensive service to the whole population of the United Kingdom.^22^ We also considered the Townsend Deprivation Index, with higher values representing a lower SES.^23^

The total household income (in pounds) before tax was investigated using baseline questionnaires. The potential responses included less than £18 000 (<$22 975), £18 000 to £30 999 ($22 975-$39 567), £31 000 to £51 999 ($39 567-$66 372), £52 000 to £100 000 ($66 372-$127 640), and >£100 000 (>$127 640) (currencies are based on 2019 rates), and “Do not know” or “Prefer not to answer” (coded as missing and thus excluded from assessment). Education qualifications were grouped into the categories of higher education (college or university degree, other professional qualifications) and others.^24^ Occupation was categorized as employed (in paid employment or self-employed, retired, performing unpaid or voluntary work, or being full or part-time students) and others.^21^

### Phenotypic Age and Age Acceleration

We calculated phenotypic age to measure the aging process and discrepancy of aging compared with chronological age.^25^ Briefly, a Cox proportional hazard elastic net model based on 10-fold cross-validation was applied to select biomarkers associated with mortality risk.^25^ The selected biomarkers were obtained from biological samples of participants enrolled at baseline. These samples were processed in the UK Biobank central laboratory within 24 hours of drawing blood with a Beckman Coulter LH750 instrument.^26^

Using chronological age and biomarkers, we calculated phenotypic age using a parametric proportional hazards model based on the Gompertz distribution.^2,3^ Details of the phenotypic age calculation and included biomarkers are shown in the eMethods and eTable 1 in Supplement 1. We further calculated the phenotypic age discrepancy as the residual by fitting a linear regression model with phenotypic age as the response variable and chronological age as the explanatory variable.^3^ A positive phenotypic age discrepancy value represents an individual who was biologically older, while a negative phenotypic age discrepancy value represents a biologically younger individual.

### Ascertainment of Mortality

The death date and causes of death in the UK Biobank were obtained from death certificates held within the National Health Service Information Centre (England and Wales) and the National Health Service Central Register Scotland (Scotland). Causes of death were identified using the *International Statistical Classification of Diseases and Related Health Problems, Tenth Revision *codes (eTable 2 in Supplement 1).^27^ Cause-specific mortality included cardiovascular disease (CVD) mortality and cancer mortality.^28^

### Covariates

We selected covariates for adjustment, including sociodemographic characteristics, lifestyles, and prevalent major chronic diseases (diabetes, hypertension, chronic kidney disease, cardiovascular disease, and cancer), as previously reported.^3,12,13^ We also considered histories of bilateral oophorectomy and hysterectomy because these conditions are considered related to an increased risk of menopausal vasomotor symptoms and neurocognitive aging.^29,30^ The sociodemographic characteristics included ethnic background, education, and other SES indicators. In UK Biobank, ethnic background is self-reported via touchscreen questionnaire. The original ethnicity categories were Asian or Asian British, Black or Black British, Chinese, multiracial, White, and other ethnic group. We grouped ethnicity as 2 categories: White and other, which included Asian or Asian British, Black or Black British, Chinese, and multiracial individuals as well as those who selected other ethnic group. Lifestyle factors included physical activity and tobacco and nicotine exposure. We considered tobacco and nicotine exposure because previous studies have shown that smoking, including second-hand smoke, leads to elevated risks of morbidity and mortality in women^31^ as well as aging.^32^ We defined tobacco and nicotine exposure following the latest Life’s Essential 8 metric recommended by the American Heart Association.^33^ Details of the metric construction are shown in eTable 3 in Supplement 1.

### Statistical Analysis

The mean (SD) or median (IQR) was used to describe continuous variables, and numbers and percentages were used for categorical variables. Differences between SES groups were tested using the Student *t* test, χ^2^ test, or Wilcoxon rank test.

We used multivariable linear regression models to assess associations of HT use, including whether HT was used, the age of starting HT, the duration of HT use, with phenotypic age discrepancy. We also conducted stratified analyses by SES indicators and applied least square means to estimate phenotypic age discrepancy values of different combinations of HT use and SES indicators. We then examined the interactions between HT use and SES variables and other baseline covariates.

We conducted a mediation analysis to examine whether the phenotypic age discrepancy mediates the associations between HT use and mortality. The mediation analysis was conducted using the difference method, with phenotypic age discrepancy modeled as the hypothetical mediating variable. The difference method, which was applied using the public %MEDIATE SAS macro, has been previously described.^21,34^

Several sensitivity analyses were conducted. First, we excluded individuals with bilateral oophorectomy or hysterectomy at baseline. Second, we further retained individuals currently using HT and repeated the primary analysis by treating current HT use as a single category. We also reanalyzed the associations between the age of starting HT and its duration and biological aging discrepancy in current HT users. Third, to address reverse causation, we excluded participants who completed the biological aging assessment within 1 year of the baseline survey. The date of the biological aging assessment was determined as the date when the participants completed the collection of biological samples. Fourth, to depict the exposure-response association pattern, restricted cubic spline modeling techniques were applied and restricted to individuals who had ever used HT. Fifth, in addition to restricted cubic spline modeling, segmented regression analysis was conducted, with the Davies test applied to examine the location of the turning point.^35^ We also excluded women with an early menopause age (<44 years) and repeated the segmented analysis. Sixth, we examined the exposure-response associations between the phenotypic age discrepancy and mortality risk. Finally, to evaluate the selection bias, a nonresponse analysis was conducted comparing baseline characteristics of included and excluded participants.

Statistical analysis was conducted using SAS version 9.4 (SAS Institute) and R language 4.3.1 (R Project for Statistical Computing). A 2-tailed α = .05 was considered statistically significant.

## Results

Among the 117 763 postmenopausal women in the UK Biobank (mean [SD] age, 60.2 [5.4] years), 47 461 (40.3%) had ever used HT (Table 1). The mean (SD) phenotypic age of the whole population was 52.1 (7.9) years. Individuals who had ever used HT were older in chronological and phenotypic age and less educated, and they had a lower income, higher exposure to nicotine, more prevalent chronic diseases, and higher proportions of bilateral oophorectomy and hysterectomy than those who never used HT.

**Table 1.** Baseline Characteristics of Participants According to Hormone Therapy History

Characteristic	Participants, No. (%)			*P* value^a^
	Total (n = 117 763)	HT		
		Never use (n = 70 302)	Ever use (n = 47 461)	
Chronological age, mean (SD), y	60.2 (5.4)	59.3 (5.6)	61.6 (4.7)	<.001
Phenotypic age, mean (SD), y	52.1 (7.9)	51.2 (8.2)	53.5 (7.3)	<.001
Ethnicity				
White	112 498 (95.5)	66 354 (94.4)	46 144 (97.2)	<.001
Other^b^	5265 (4.5)	3948 (5.6)	1317 (2.8)	
Higher education	52 945 (45.0)	32 425 (46.1)	20 520 (43.2)	<.001
Annual household income ≥£31 000^c^	39 352 (33.4)	24 981 (35.5)	14 371 (30.3)	<.001
Employed	109 583 (93.1)	64 835 (92.2)	44 748 (94.3)	<.001
Townsend Deprivation Index below median (−2.30)	58 824 (50.0)	34 678 (49.3)	24 146 (50.9)	<.001
Moderate-to-vigorous physical activity ≥150 min/wk	84 278 (71.6)	49 588 (70.5)	34 690 (73.1)	<.001
Tobacco and nicotine exposure score, mean (SD)	79.5 (30.3)	81.0 (29.8)	77.2 (30.8)	<.001
Chronic kidney disease	3775 (3.2)	2067 (2.9)	1708 (3.6)	<.001
Diabetes	5682 (4.8)	3510 (5.0)	2172 (4.6)	<.001
Hypertension	66 446 (56.4)	38 476 (54.7)	27 970 (58.9)	<.001
Cardiovascular disease	6980 (5.9)	3643 (5.2)	3337 (7.0)	<.001
Cancer	16 554 (14.1)	9635 (13.7)	6919 (14.6)	<.001
Bilateral oophorectomy	6065 (5.2)	1916 (2.7)	4149 (8.7)	<.001
Hysterectomy	12 715 (10.8)	4572 (6.5)	8143 (17.2)	<.001
Hormone therapy usage, median (IQR), y				
Age started hormone therapy	49.0 (45.0-51.0)	NA	49.0 (45.0-51.0)	NA
Age last used hormone therapy	55.0 (51.0-58.0)	NA	55.0 (51.0-58.0)	NA
Duration of hormone therapy	5.0 (2.0-10.0)	NA	5.0 (2.0-10.0)	NA

Abbreviations: HT, hormone therapy; NA, not applicable.

^a^ Group differences were compared using Student *t* test, χ^2^ test, or Wilcoxon rank test.

^b^ The other group included Asian or Asian British, Black or Black British, Chinese, and multiracial individuals as well as those who selected other ethnic group on the questionnaire.

^c^ To convert from pounds to 2019 US dollars, multiply by 1.28.

After controlling for other factors, having ever used HT was associated with 0.17 (95% CI, 0.10-0.23) fewer years of aging discrepancy (Table 2). Starting HT after age 45 years was associated with a smaller aging discrepancy, while a larger aging discrepancy was observed in those who started HT before age 44 years compared with individuals who never used HT. In individuals with HT use of any duration, we observed a smaller aging discrepancy, especially in those who used HT for 4 to 8 years.

**Table 2.** Associations Between HT and Phenotypic Age Discrepancy

HT	No. of participants	β (95% CI)^a^	*P* value
History of hormone therapy			
Never use HT	70 302	0 [Reference]	NA
Ever use HT	47 461	−0.17 (−0.23 to −0.10)	<.001
Age started hormone therapy			
Never use HT	70 302	0 [Reference]	NA
≤44 y	8899	0.33 (0.21 to 0.44)	<.001
45-49 y	16 485	−0.20 (−0.29 to −0.11)	<.001
50-54 y	17 908	−0.32 (−0.41 to −0.24)	<.001
≥55 y	4169	−0.32 (−0.48 to −0.15)	<.001
Duration of hormone therapy			
Never use HT	70 302	0 [Reference]	NA
≤1 y	10 566	−0.11 (−0.22 to −0.01)	.04
>1-4 y	9910	−0.18 (−0.29 to −0.07)	.002
>4-8 y	12 745	−0.25 (−0.35 to −0.15)	<.001
>8 y	12 240	−0.12 (−0.22 to −0.02)	.02

Abbreviations: HT, hormone therapy; NA, not applicable.

^a^ Coefficients were derived using multivariable linear regression models, with positive values representing older biological age and negative values otherwise. Covariates included ethnicity, education, physical activity, tobacco and nicotine exposure, diabetes, hypertension, chronic kidney disease, cardiovascular disease, bilateral oophorectomy, and hysterectomy.

**Figure 1.**
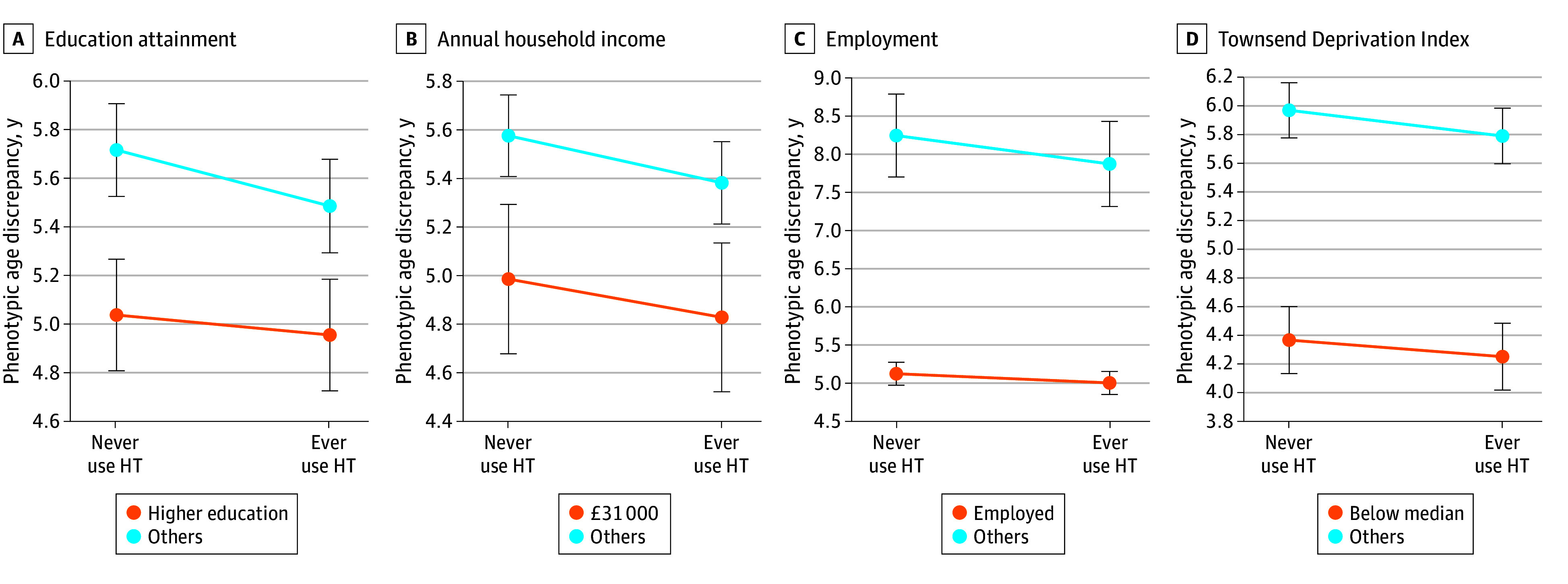
Estimated Phenotypic Age Discrepancy by Hormone Therapy (HT) and Socioeconomic Status Estimates were derived as least square means using multivariable linear regression models, with higher values representing expanded aging discrepancy. Covariates were identical to the models in Table 2. Points represent point estimates, and bars represent 95% CIs. The median Townsend Deprivation Index score was −2.30. To convert from pounds to 2019 US dollars, multiply by 1.28.

**Figure 2.**
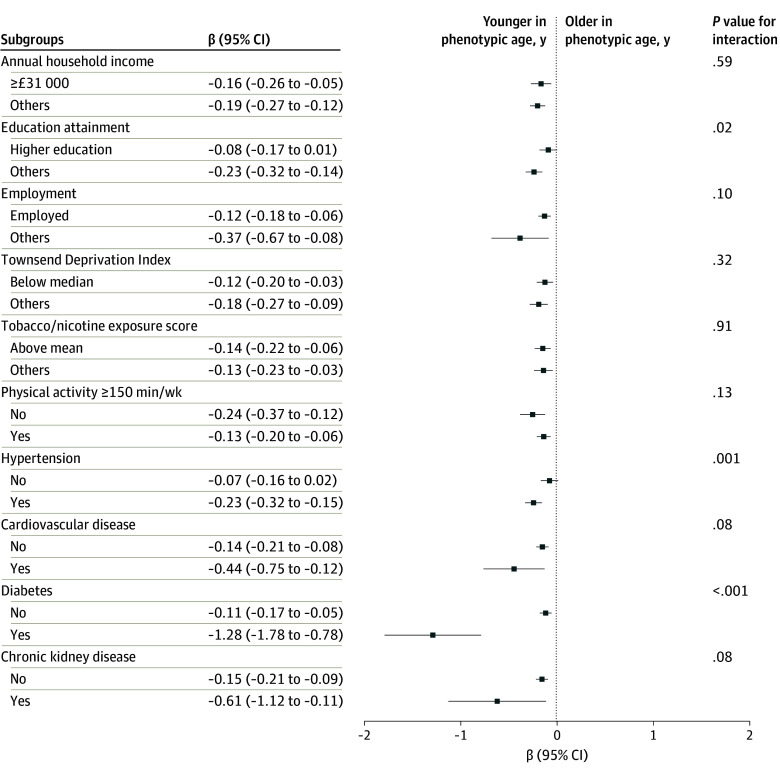
Subgroup Analysis of Associations Between Hormone Therapy and Phenotypic Age Discrepancy Coefficients were derived using multivariable linear regression models, with positive values representing older biological age and negative values otherwise. Covariates were identical to the models in Table 2. Subgroups were defined according to socioeconomic status indicators and baseline covariates. The median Townsend Deprivation Index score was −2.30. To convert from pounds to 2019 US dollars, multiply by 1.28.

HT users had a smaller aging discrepancy than those who never used HT, regardless of SES (Figure 1). Moreover, individuals with a lower SES had a larger aging discrepancy, regardless of HT use. For example, among participants with higher education, those who never used HT and ever used HT had aging discrepancies of 5.03 (95% CI, 4.80 to 5.26) years and 4.95 (95% CI, 4.72 to 5.18) years, respectively. Among participants with other education, the aging discrepancies in individuals who never used HT and ever used HT were 5.71 (95% CI, 5.52 to 5.90) and 5.48 (95% CI, 5.29 to 5.67) years, respectively. HT use was associated with a smaller aging discrepancy among all subgroups (Figure 2). Significant interactions were observed between HT use and education (higher education: β, −0.08 [95% CI, −0.17 to 0.01]; other education: β, −0.23 [95% CI, −0.32 to −0.14]; *P* for interaction = .02), hypertension (no hypertension: β, −0.07 [95% CI, −0.16 to 0.02]; hypertension: β, −0.23 [95% CI, −0.32 to −0.15]; *P* for interaction = .001), and diabetes (no diabetes: β, −0.11 [95% CI, −0.17 to −0.05]; diabetes: β, −1.28 [95% CI, −1.78 to −0.78]; *P* for interaction < .001).

**Figure 3.**
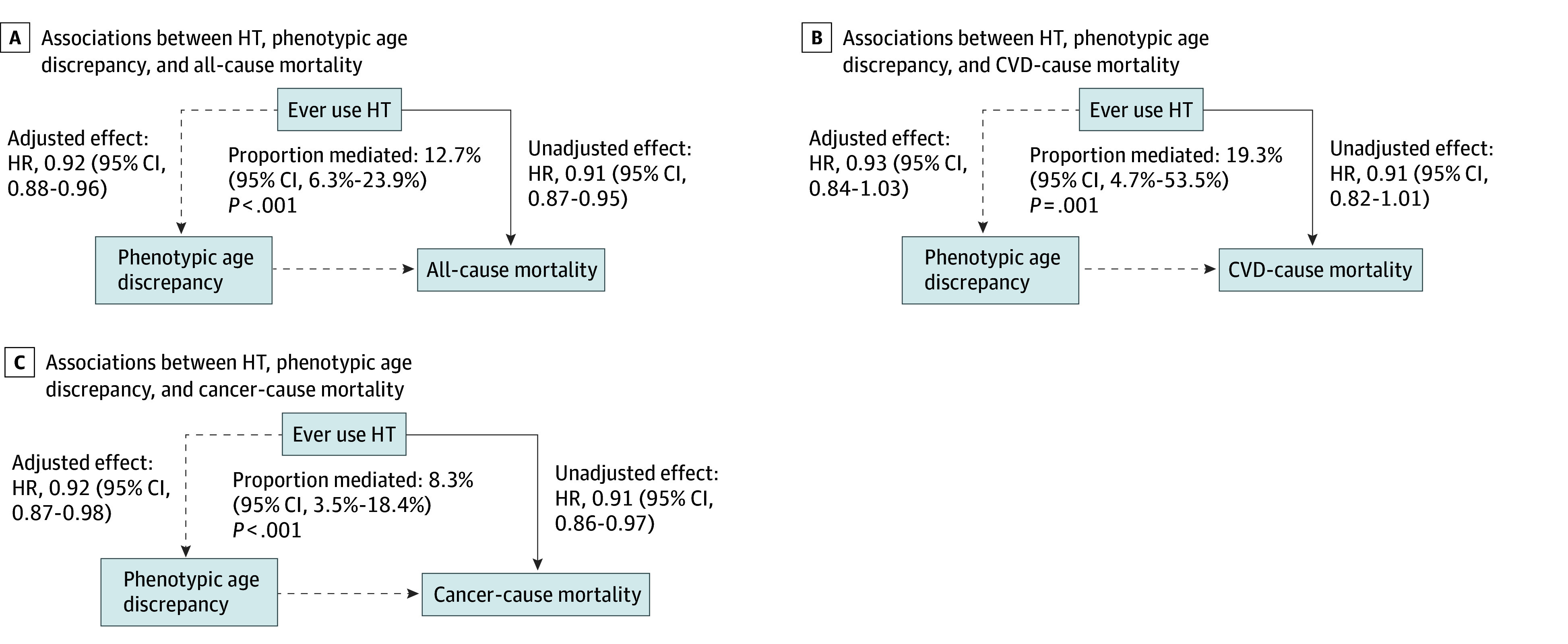
Mediation Analysis of Associations Between Hormone Therapy (HT), Phenotypic Age Discrepancy, and Mortality The hazard ratio (HR) was calculated using the Cox proportional hazard regression models, adjusting for ethnicity, education, physical activity, tobacco and nicotine exposure, diabetes, hypertension, chronic kidney disease, cardiovascular disease, cancer, bilateral oophorectomy, and hysterectomy. The mediation analysis was conducted using the difference method by comparing the effect before and after adjusting for the hypothesized mediator (phenotypic age discrepancy). The unadjusted effect represents the estimate from the Cox regression without adjusting for the hypothesized mediator, while the adjusted effect comes from the Cox regression adjusting for the hypothesized mediator. CVD indicates cardiovascular disease.

The phenotypic age discrepancy significantly mediated the association between HT use and mortality risk (Figure 3). Regarding all-cause mortality, 12.7% (95% CI, 6.3%-23.9%) of observed associations were explained by the association between HT and phenotypic age discrepancy. Regarding CVD and cancer mortality, the mediated percentages were 19.3% (95% CI, 4.7%-53.5%) and 8.3% (95% CI, 3.5%-18.4%), respectively.

In sensitivity analyses, the magnitude of associations was not altered after excluding individuals with bilateral oophorectomy (eTable 4 in Supplement 1) or hysterectomy (eTable 5 in Supplement 1). Currently using HT was associated with 0.56 (95% CI, 0.42 to 0.69) more years of aging discrepancy, while ever using HT was associated with smaller aging discrepancy compared with never using HT (eTable 6 in Supplement 1). When restricted to current HT users, HT starting at an age 45 years or older was associated with a smaller aging discrepancy compared with starting HT before 44 years (eTable 7 in Supplement 1). The median duration of HT use in current HT users was 9.0 (IQR, 4.0-14.0) years, which was longer than that in individuals who had ever used HT (5.0 [IQR, 2.0-10.0] years) (*P* < .001) (eTable 8 in Supplement 1). We found that these associations remained robust after excluding individuals who completed outcome assessment within 1 year of the baseline survey (eTable 9 in Supplement 1). Significant nonlinear patterns of associations were observed between the age of starting HT and the duration of HT use and phenotypic age, with a J-shaped curve identified (eFigure 2 in Supplement 1). Clear breakpoints were observed by segmented regression (eFigure 3 in Supplement 1). Regarding the age of starting HT, the estimated breakpoint was 48.4 (95% CI, 47.2-49.5) years, before which HT was negatively associated with aging discrepancy (eTable 10 in Supplement 1). Regarding the duration of HT, the estimated breakpoint was 7.4 (95% CI, 6.0-8.9) years, after which HT use was positively associated with aging discrepancy (eTable 10 in Supplement 1). A similar pattern was observed after excluding women with early menopause (eTable 11 in Supplement 1). The larger aging discrepancy was consistently associated with increased mortality (eFigure 4 in Supplement 1). The nonresponse analysis (eTable 12 in Supplement 1) showed that excluded postmenopausal women were older, less educated, and had a lower income and more prevalent chronic diseases than included participants.

## Discussion

This large population-based, retrospective cohort study found a significant association between historical HT use and a smaller biological aging discrepancy in postmenopausal women. Furthermore, this association was modified by SES and more evident in women with a disadvantaged socioeconomic position. In addition, the mediation analysis showed that the phenotypic age discrepancy significantly mediated the association between ever using HT and decreased mortality risk, especially for CVD mortality. To our knowledge, this is the first study to investigate the association between HT use and biological aging discrepancy and to examine the modifying role of the SES.

The beneficial effects of HT on other conditions have been previously reported. Hodis et al^36^ reported that in women who initiated HT at younger than 60 years and/or fewer than 10 years since menopause, the risks of all-cause mortality and coronary heart disease were reduced by 30% to 50%.^36^ This finding suggested a more effective strategy than other primary CVD prevention measures. Furthermore, several studies have shown additional benefits of HT, including protection against new-onset diabetes,^37^ nonvertebral fractures, and osteoporosis-related fractures in women younger than 60 years.^38,39^ Our study may offer a new perspective regarding the potential health benefits of HT use because of the continually increasing demand for therapy for postmenopausal women.

Globally, the aging population is growing at an unprecedented pace, causing new challenges in various aspects of society.^40^ Preventing diseases and improving health in the rapidly aging population requires efforts to target delaying aging.^41^ In addition, measuring the rate of aging has the potential to capture population heterogeneity in aging.^42^ In our study, using HT for 4 to 8 years was associated with 0.25 fewer years of biological aging discrepancy. In a previous study, middle-aged adults with 1 major chronic disease were an average of 0.2 years older in phenotypic age than disease-free counterparts.^25^ Moreover, each 1-year increment in phenotypic age (adjusted for chronological age) was associated with as much as a 9% higher all-cause and a 20% higher cause-specific mortality risk.^25^ Accordingly, the 0.25 years of delayed aging observed in our study could translate to approximately 2.25% decreased risk of all-cause mortality and 5% decreased risk of cause-specific mortality. Therefore, the observed magnitude of associations in our study could be relevant for current clinical practice.

We also found that the association between historical HT use and phenotypic age discrepancy explained 8.3% to 19.3% of the associations between HT and mortality. This finding not only facilitates interpretation of the effect of HT on all-cause mortality risk by 20% to 40%,^36,43,44,45^ but it also highlights the importance of measuring phenotypic age for preventing early mortality risk in postmenopausal women. Despite the potential benefits of HT, several previous studies reported that HT was associated with an increased risk of breast cancer and dementia.^46,47^ HT is a recommended therapy for postmenopausal women and is used in a large proportion of the population. Therefore, further investigations are warranted to confirm our main findings and evaluate the risks and benefits of HT.

We also found that historical HT use and a smaller aging discrepancy were more strongly associated in women with disadvantaged SES than in those with higher SES. A previous study showed that higher SES was associated with slower aging in all measures, including phenotypic age.^48^ In line with this previous study, we found that individuals with higher SES had a smaller aging discrepancy, regardless of HT use. However, the findings also could be interpreted as an advantaged position regarding various health aspects in individuals with high SES. Therefore, this population might not benefit from using HT. Nevertheless, our findings suggest the importance of promoting HT use in those with disadvantaged SES.

Another notable finding was the potential threshold effects between the age of starting HT and duration of HT use and phenotypic aging. The association between historical HT use and a smaller aging discrepancy was more prominent before age 48.4 years. Furthermore, historical HT use was associated with a smaller aging discrepancy within 7.4 years, while an inverse association was found for HT use lasting longer than 7.4 years. A categorical analysis yielded similar results. Similarly, an observational study showed that introducing HT in midlife (age 48.7 years) was associated with a 26% reduced risk of dementia compared with a 48% increased risk when HT was initiated in later life (age 76 years).^16^ Saleh et al^17^ reported that the role of HT initiation timing was a mediator of the cognitive effect of HT use. Two meta-analyses of randomized clinical trials^49,50^ reported that HT reduced all-cause mortality by 39% and incident coronary heart disease by 32% when initiated in women younger than 60 years and/or with fewer than 10 years since menopause.^49,50^ Overall, our finding regarding the timing of initiating HT is consistent with previous studies, highlighting the importance of thorough consideration in developing HT prescription regimes to maximize the potential health benefits. The timing hypothesis could also explain our findings that current HT users had a larger aging discrepancy, with a significantly longer HT duration than historical HT users. These findings indicate a possible detrimental effect due to excessive duration of HT.

The abnormal menopause process in women who initiated HT before 48 years of age could be a key mechanism in explaining our findings. Xu et al^51^ reported that women with premature menopause had twice the odds of experiencing multimorbidity by age 60 years and 3 times the odds of developing multimorbidity at all. Szeliga et al^52^ reported that autoimmune processes account for 4% to 30% of premature menopause.^52^ Women who initiate HT before age 48 years might experience premature menopause. Therefore, they might be a distinct population with chronic conditions or comorbidities that are not present in women undergoing natural menopause, leading to different outcomes regarding age discrepancy.

The present study has several strengths, namely the large sample size and novel aging discrepancy measures. Various sensitivity analyses were conducted and supported the robustness of the major findings.

### Limitations

We acknowledge some important limitations. First, the use of HT information was evaluated using self-reported data, which could have been subject to measurement bias. Second, our study solely considered biological age measurements at a single point in time and lacked methods of capturing long-term biological aging trajectory. Third, the current analysis failed to account for the dose and route of HT owing to data restrictions, which could be an important limitation affecting the validity of our findings. Fourth, most of the UK Biobank participants were White, thus restricting the generalization of our findings to other ethnicities. Fifth, HT use and biological aging were assessed at baseline, which caused a challenge of reverse causation. Although our findings remained robust in the sensitivity analysis, causations should be interpreted with caution. Additionally, because of the nature of observational studies, we could not eliminate the effect of residual confounding, which impedes further steps toward conclusively defining causal relationships. Therefore, further studies that take these challenges into account will need to be undertaken.

## Conclusions

In this large population-based, retrospective cohort study, we found that postmenopausal women who historically received HT were biologically younger than those who did not receive HT, regardless of socioeconomic background. Our findings highlight the importance of emphasizing HT use in postmenopausal women to promote inclusive healthy aging. Further investigations are warranted to confirm HT’s clinical benefits.

## Notes

Supplement 1.
eMethods.

eReferences.
**eTable 1.** Variables and Gompertz Coefficients Used for Calculating the Phenotypic Aging Measures in the UK Biobank **eTable 2.** *International Classification of Diseases *(*ICD*) Codes Used for Identification of Cause-Specific Mortality **eTable 3.** Scoring of the Tobacco and Nicotine Exposure According to Life’s Essential 8 Cardiovascular Health (CVH) Metrics in the UK Biobank **eTable 4.** Associations Between Hormone Therapy and Phenotypic Age Discrepancy, Excluding Individuals With Bilateral Oophorectomy **eTable 5.** Associations Between Hormone Therapy and Phenotypic Age Discrepancy, Excluding Individuals With Hysterectomy **eTable 6.** Associations Between Hormone Therapy and Phenotypic Age Discrepancy, Further Accounting for Individuals Currently Using HT **eTable 7.** Associations Between Hormone Therapy and Phenotypic Age Discrepancy, Restricted to Individuals Currently Using HT **eTable 8.** Hormone Therapy Usage Characteristics Among Individuals Ever and Currently Using HT **eTable 9.** Associations Between Hormone Therapy and Phenotypic Age Discrepancy, Excluding Individuals Completing Outcome Assessment Within 1 Year of Baseline Survey **eTable 10.** Segmented Regression Assessing Associations Between Hormone Therapy and Phenotypic Age Discrepancy **eTable 11.** Segmented Regression Assessing Associations Between Hormone Therapy and Phenotypic Age Discrepancy, Excluding Women With Early Menopause **eTable 12.** Nonresponse Analysis Comparing Baseline Characteristics of Included and Excluded Postmenopausal Women Participants in the UK Biobank **eFigure 1.** Participant Selection Diagram **eFigure 2.** Dose-Response Associations Between Age Started and Years Used Hormone Therapy and Phenotypic Age and Phenotypic Age Discrepancy **eFigure 3.** Segmented Regression Assessing Dose-Response Associations Between Age Started and Years Used Hormone Therapy and Phenotypic Age Discrepancy **eFigure 4.** Dose-Response Associations Between Phenotypic Age Discrepancy and All-Cause and Cause-Specific Mortality

Supplement 2.
Data Sharing Statement
