# Supplementary material for: Hormone Therapy and Biological Aging in Postmenopausal Women
Source: JAMA Netw Open. 2024 Aug 29;7(8):e2430839. doi: 10.1001/jamanetworkopen.2024.30839 (PMC11362863; doi:10.1001/jamanetworkopen.2024.30839)
Supplement: Supplement 1. — eMethods. eReferences. eTable 1. Variables and Gompertz Coefficients Used for Calculating the Phenotypic Aging Measures in the UK Biobank eTable 2. International Classification of Diseases (ICD) Codes Used for Identification of Cause-Specific Mortality eTable 3. Scoring of the Tobacco and Nicotine Exposure According to Life’s Essential 8 Cardiovascular Health (CVH) Metrics in the UK Biobank eTable 4. Associations Between Hormone Therapy and Phenotypic Age Discrepancy, Excluding Individuals With Bilateral Oophorectomy eTable 5. Associations Between Hormone Therapy and Phenotypic Age Discrepancy, Excluding Individuals With Hysterectomy eTable 6. Associations Between Hormone Therapy and Phenotypic Age Discrepancy, Further Accounting for Individuals Currently Using HT eTable 7. Associations Between Hormone Therapy and Phenotypic Age Discrepancy, Restricted to Individuals Currently Using HT eTable 8. Hormone Therapy Usage Characteristics Among Individuals Ever and Currently Using HT eTable 9. Associations Between Hormone Therapy and Phenotypic Age Discrepancy, Excluding Individuals Completing Outcome Assessment Within 1 Year of Baseline Survey eTable 10. Segmented Regression Assessing Associations Between Hormone Therapy and Phenotypic Age Discrepancy eTable 11. Segmented Regression Assessing Associations Between Hormone Therapy and Phenotypic Age Discrepancy, Excluding Women With Early Menopause eTable 12. Nonresponse Analysis Comparing Baseline Characteristics of Included and Excluded Postmenopausal Women Participants in the UK Biobank eFigure 1. Participant Selection Diagram eFigure 2. Dose-Response Associations Between Age Started and Years Used Hormone Therapy and Phenotypic Age and Phenotypic Age Discrepancy eFigure 3. Segmented Regression Assessing Dose-Response Associations Between Age Started and Years Used Hormone Therapy and Phenotypic Age Discrepancy eFigure 4. Dose-Response Associations Between Phenotypic Age Discrepancy and All-Cause and Cause-Specific Mort [file jamanetwopen-e2430839-s001.pdf]

Supplemental Online Content

Liu Y, Li C. Hormone Therapy and Biological Aging in Postmenopausal Women. *JAMA Netw Open.* 2024;7(8):e2430839. doi:10.1001/jamanetworkopen.2024.30839

eMethods.

eReferences.

eTable 1. Variables and Gompertz Coefficients Used for Calculating the Phenotypic Aging Measures in the UK Biobank

eTable 2. International Classification of Diseases (ICD) Codes Used for Identification of Cause-Specific Mortality

eTable 3. Scoring of the Tobacco and Nicotine Exposure According to Life's Essential 8 Cardiovascular Health (CVH) Metrics in the UK Biobank

eTable 4. Associations Between Hormone Therapy and Phenotypic Age Discrepancy, Excluding Individuals With Bilateral Oophorectomy

eTable 5. Associations Between Hormone Therapy and Phenotypic Age Discrepancy, Excluding Individuals With Hysterectomy

eTable 6. Associations Between Hormone Therapy and Phenotypic Age Discrepancy, Further Accounting for Individuals Currently Using HT

eTable 7. Associations Between Hormone Therapy and Phenotypic Age Discrepancy, Restricted to Individuals Currently Using HT

eTable 8. Hormone Therapy Usage Characteristics Among Individuals Ever and Currently Using HT

eTable 9. Associations Between Hormone Therapy and Phenotypic Age Discrepancy, Excluding Individuals Completing Outcome Assessment Within 1 Year of Baseline Survey

eTable 10. Segmented Regression Assessing Associations Between Hormone Therapy and Phenotypic Age Discrepancy

eTable 11. Segmented Regression Assessing Associations Between Hormone Therapy and Phenotypic Age Discrepancy, Excluding Women With Early Menopause

eTable 12. Nonresponse Analysis Comparing Baseline Characteristics of Included and Excluded Postmenopausal Women Participants in the UK Biobank

eFigure 1. Participant Selection Diagram

eFigure 2. Dose-Response Associations Between Age Started and Years Used

© 2024 Liu et al. *JAMA Network Open.*

Hormone Therapy and Phenotypic Age and Phenotypic Age Discrepancy

eFigure 3. Segmented Regression Assessing Dose-Response Associations Between Age Started and Years Used Hormone Therapy and Phenotypic Age Discrepancy

eFigure 4. Dose-Response Associations Between Phenotypic Age Discrepancy and All-Cause and Cause-Specific Mortality

This supplemental material has been provided by the authors to give readers additional information about their work.

© 2024 Liu et al. *JAMA Network Open.*

eMethods

1. Calculation of the Phenotypic Age in the UK Biobank

Referenced from previous studies, we calculated the Phenotypic Age using chronological age and 9 biomarkers (albumin, creatinine, glucose, [log-transformed] C-reactive protein, lymphocyte percent, mean cell volume, red blood cell distribution width, alkaline phosphatase, and white blood cell count).[1,2] A parametric proportional hazards model based on the Gompertz distribution was applied to formulate the equation for calculating the Phenotypic Age, presented as follows:

$$Phenotypic\ Age = 141.50225 + \frac{\ln[-0.00553 \times \ln(1 - mortality\ risk)]}{0.090165}$$

Where

$$mortality\ risk = 1 - e^{-e^{xb}[\exp(120\times\gamma)-1]/\gamma}$$

$$\gamma = 0.0076927$$

$$xb = -19.9067 - 0.0336 \times \text{albumin} + 0.0095 \times \text{creatinine} + 0.1953 \times \text{glucose} + 0.0954 \times \ln(C\text{–}reactive\ protein) - 0.0120 \times lymphocyte\ percentage + 0.0268 \times mean\ cell\ volume + 0.3306 \times red\ cell\ distribution\ width + 0.0019 \times alkaline\ phosphatase + 0.0554 \times white\ blood\ cell\ count + 0.0804 \times chronological\ age$$

© 2024 Liu et al. *JAMA Network Open.*

eReferences.

1. Levine ME, Lu AT, Quach A, et al. An epigenetic biomarker of aging for lifespan and healthspan. *Aging (Albany NY)*. 2018;10(4):573-591. doi:10.18632/aging.101414

2. Yang G, Cao X, Li X, et al. Association of Unhealthy Lifestyle and Childhood Adversity with Acceleration of Aging among UK Biobank Participants. *JAMA Netw Open*. 2022;(September):E2230690. doi:10.1001/jamanetworkopen.2022.30690

© 2024 Liu et al. *JAMA Network Open.*

eTable 1. Variables and Gompertz Coefficients Used for Calculating the Phenotypic Aging Measures in the UK Biobank

**Variable**	**Units**	**Weight**	**UK Biobank Data-Field ID**
Albumin	g/L	-0.0336	30600
Creatinine	umol/L	0.0095	30700
Glucose, serum	mmol/L	0.1953	30740
C-reactive protein (log-transformed)	mg/dL	0.0954	30710
Lymphocyte percent	%	-0.0120	30180
Mean cell volume	fL	0.0268	30270
Red cell distribution width	%	0.3306	30070
Alkaline phosphatase	U/L	0.0019	30610
White blood cell count	10^9 cells/L or 10^3 cells/uL	0.0554	30000
Chronological age	Years	0.0804	34, 53
Constant		-19.9067	
Gamma		0.0076927	

© 2024 Liu et al. *JAMA Network Open.*

eTable 2. International Classification of Diseases (ICD) Codes Used for Identification of Cause-Specific Mortality

**Cause of Death**	**ICD-10 Codes**
Cancer	C00-C97
Cardiovascular Disease	I00-I99

© 2024 Liu et al. *JAMA Network Open.*

eTable 3. Scoring of the Tobacco and Nicotine Exposure According to Life's Essential 8 Cardiovascular Health (CVH) Metrics in the UK Biobank

CVH metric	Method of measurement	Points	Scoring (individual)	UK Biobank Data-Field ID [a]
Tobacco/nicotine exposure score				
	Self-reported tobacco use (current smoking status and history of smoking) or secondhand smoke exposure (Participants were asked by "Does anyone in your household smoke". Secondhand smoke exposure was defined as if participants' responses are "yes, one household member smokes" or "Yes, more than one household member smokes").	100<br>75<br>50<br>25<br>0	Never smoker<br>Former smoker, quit ≥ 5 years<br>Former smoker, quit 1–<5 years<br>Former smoker, quit <1 year<br>Current smoker	20116, 2897, 6194, 1249, 1259, 1269
		Subtract 20 points (unless score is 0) for living household with active indoor smoker in home. "Smoked occasionally in the past" was regarded as "Former smoker, quit 1–<5 years"; "Just tried once or twice in the past" was regarded as "Former smoker, quit ≥ 5 years".		

[a] UK Biobank Data-Field ID can be used to search related variables for scoring.

© 2024 Liu et al. *JAMA Network Open.*

eTable 4. Associations Between Hormone Therapy and Phenotypic Age Discrepancy, Excluding Individuals With Bilateral Oophorectomy

**Hormone therapy**	**No. of participants**	**β (95% CI)** [a]	***P* value**
History of hormone therapy			
Never use HT	68,386	0 [Reference]	NA
Ever use HT	43,312	-0.16 (-0.22 to -0.10)	<0.001
Age started hormone therapy			
Never use HT	68,386	0 [Reference]	NA
≤ 44 years	7668	0.35 (0.22 to 0.47)	<0.001
45-49 years	15,077	-0.17 (-0.27 to -0.08)	<0.001
50-54 years	16,638	-0.33 (-0.42 to -0.24)	<0.001
≥ 55 years	3929	-0.34 (-0.50 to -0.17)	<0.001
Duration of hormone therapy			
Never use HT	68,386	0 [Reference]	NA
≤ 1 years	9872	-0.11 (-0.22 to 0.00)	0.058
>1-4 years	9270	-0.19 (-0.30 to -0.07)	<0.001
>4-8 years	11,727	-0.24 (-0.35 to -0.14)	<0.001
> 8 years	12,443	-0.10 (-0.20 to 0.00)	0.056

HT, hormone therapy; CI, confidence interval; NA: not applicable.

[a] Coefficients were derived using multivariable linear regression models, with positive values representing older in biological age and negative values otherwise. Covariates included ethnic background, education, physical activity, tobacco/nicotine exposure, diabetes, hypertension, chronic kidney disease, cardiovascular disease, and hysterectomy.

© 2024 Liu et al. *JAMA Network Open.*

eTable 5. Associations Between Hormone Therapy and Phenotypic Age Discrepancy, Excluding Individuals With Hysterectomy

**Hormone therapy**	**No. of participants**	**β (95% CI)** [a]	***P* value**
History of hormone therapy			
Never use HT	65,730	0 [Reference]	NA
Ever use HT	39,318	-0.18 (-0.25 to -0.11)	<0.001
Age started hormone therapy			
Never use HT	65,730	0 [Reference]	NA
≤ 44 years	6491	0.32 (0.18 to 0.45)	<0.001
45-49 years	13,678	-0.19 (-0.28 to -0.09)	<0.001
50-54 years	15,475	-0.34 (-0.43 to -0.24)	<0.001
≥ 55 years	3674	-0.35 (-0.53 to -0.18)	<0.001
Duration of hormone therapy			
Never use HT	65,730	0 [Reference]	NA
≤ 1 years	9215	-0.14 (-0.25 to -0.02)	0.017
>1-4 years	8700	-0.18 (-0.30 to -0.06)	0.002
>4-8 years	10,715	-0.27 (-0.38 to -0.16)	<0.001
> 8 years	10,688	-0.12 (-0.23 to -0.01)	0.026

HT, hormone therapy; CI, confidence interval; NA: not applicable.

[a] Coefficients were derived using multivariable linear regression models, with positive values representing older in biological age and negative values otherwise. Covariates included ethnic background, education, physical activity, tobacco/nicotine exposure, diabetes, hypertension, chronic kidney disease, cardiovascular disease, and bilateral oophorectomy.

© 2024 Liu et al. *JAMA Network Open.*

eTable 6. Associations Between Hormone Therapy and Phenotypic Age Discrepancy, Further Accounting for Individuals Currently Using HT

**Hormone therapy**	**No. of participants**	**β (95% CI)** [a]	***P* value**
Overall hormone therapy status			
Never use HT	70,302	0 [Reference]	NA
Ever use HT	47,461	-0.16 (-0.23 to -0.10)	<0.001
Currently use HT	6908	0.56 (0.42 to 0.69)	<0.001
Whether exposed to hormone therapy			
Never use HT	70,302	0 [Reference]	NA
Ever or currently use HT	54,369	-0.07 (-0.13 to -0.01)	0.018
History of hormone therapy			
Never or currently use HT	77,210	0 [Reference]	NA
Ever use HT	47,461	-0.22 (-0.28 to -0.16)	<0.001
Age started hormone therapy			
Never use HT	70,302	0 [Reference]	NA
≤ 44 years	8899	0.33 (0.21 to 0.45)	<0.001
45-49 years	16,485	-0.20 (-0.29 to -0.11)	<0.001
50-54 years	17,908	-0.32 (-0.41 to -0.23)	<0.001
≥ 55 years	4169	-0.32 (-0.48 to -0.15)	<0.001
Currently use HT	6908	0.56 (0.43 to 0.69)	<0.001
Duration of hormone therapy			
Never use HT	70,302	0 [Reference]	NA
≤ 1 years	10,566	-0.11 (-0.22 to -0.00)	0.045
>1-4 years	9910	-0.18 (-0.29 to -0.07)	0.002
>4-8 years	12,745	-0.25 (-0.35 to -0.15)	<0.001
> 8 years	12,240	-0.11 (-0.21 to -0.02)	0.023
Currently use HT	6908	0.56 (0.43 to 0.69)	<0.001

HT, hormone therapy; CI, confidence interval; NA: not applicable.

[a] Coefficients were derived using multivariable linear regression models, with positive values representing older in biological age and negative

© 2024 Liu et al. *JAMA Network Open.*

values otherwise. Covariates included ethnic background, education, physical activity, tobacco/nicotine exposure, diabetes, hypertension, chronic kidney disease, cardiovascular disease, and bilateral oophorectomy.

© 2024 Liu et al. *JAMA Network Open.*

eTable 7. Associations Between Hormone Therapy and Phenotypic Age Discrepancy, Restricted to Individuals Currently Using HT

**Hormone therapy**	**No. of participants**	**β (95% CI) [a]**	***P* value**
Age started hormone therapy			
≤ 44 years	1447	0 [Reference]	NA
45-49 years	2111	-0.38 (-0.73 to -0.04)	0.029
50-54 years	2545	-0.68 (-1.02 to -0.34)	<0.001
≥ 55 years	805	-1.09 (-1.54 to -0.64)	<0.001
Duration of hormone therapy			
≤ 1 years	748	0 [Reference]	NA
>1-4 years	1089	-0.62 (-1.09 to -0.14)	0.011
>4-8 years	1459	-0.30 (-0.76 to 0.15)	0.187
> 8 years	3612	0.06 (-0.35 to 0.47)	0.780

HT, hormone therapy; CI, confidence interval; NA: not applicable.

[a] Coefficients were derived using multivariable linear regression models, with positive values representing older in biological age and negative values otherwise. Covariates included ethnic background, education, physical activity, tobacco/nicotine exposure, diabetes, hypertension, chronic kidney disease, cardiovascular disease, and bilateral oophorectomy.

© 2024 Liu et al. *JAMA Network Open.*

eTable 8. Hormone Therapy Usage Characteristics Among Individuals Ever and Currently Using HT

**Characteristics**	**Ever use HT (n=47 461)**	**Currently use HT (n=6908)**	***P* for difference [a]**
Hormone therapy usage, median [IQR], y			
Age started hormone therapy	49.0 [45.0-51.0]	49.0 [45.0-52.0]	<0.001
Age last used hormone therapy	55.0 [51.0-58.0]	58.0 [54.0-62.0]	<0.001
Duration of hormone therapy	5.0 [2.0-10.0]	9.0 [4.0-14.0]	<0.001

HT, hormone therapy; NA: not applicable.

[a] Group differences were compared using Student's t-test, chi-square test, or Wilcoxon rank test.

© 2024 Liu et al. *JAMA Network Open.*

eTable 9. Associations Between Hormone Therapy and Phenotypic Age Discrepancy, Excluding Individuals Completing Outcome Assessment Within 1 Year of Baseline Survey

**Hormone therapy**	**No. of participants**	**β (95% CI) [a]**	***P* value**
History of hormone therapy			
Never use HT	36,104	0 [Reference]	NA
Ever use HT	24,380	-0.12 (-0.20 to -0.03)	0.008
Age started hormone therapy			
Never use HT	36,104	0 [Reference]	NA
≤ 44 years	4584	0.33 (0.17 to 0.50)	<0.001
45-49 years	8469	-0.16 (-0.29 to -0.04)	0.011
50-54 years	9166	-0.24 (-0.36 to -0.12)	<0.001
≥ 55 years	2161	-0.33 (-0.56 to -0.10)	0.005
Duration of hormone therapy			
Never use HT	36,104	0 [Reference]	NA
≤ 1 years	5401	-0.05 (-0.20 to 0.10)	0.488
>1-4 years	5106	-0.14 (-0.29 to 0.01)	0.075
>4-8 years	6629	-0.21 (-0.34 to -0.07)	0.004
> 8 years	7244	-0.07 (-0.20 to 0.07)	0.334

HT, hormone therapy; CI, confidence interval; NA: not applicable.

[a] Coefficients were derived using multivariable linear regression models, with positive values representing older in biological age and negative values otherwise. Covariates included ethnic background, education, physical activity, tobacco/nicotine exposure, diabetes, hypertension, chronic kidney disease, cardiovascular disease, and bilateral oophorectomy.

© 2024 Liu et al. *JAMA Network Open.*

eTable 10. Segmented Regression Assessing Associations Between Hormone Therapy and Phenotypic Age Discrepancy

HT usage [a]	Estimated turning point (95% CI) [b]	Estimated slopes [c]		*P* for differences in slopes [d]
		Pre-turning point (95%CI)	Post-turning point (95%CI)	
Age started HT, y	48.4 (47.2, 49.5)	-0.138 (-0.158, -0.119)	-0.002 (-0.029, 0.026)	<0.001
HT usage duration, y	7.4 (6.0, 8.9)	-0.041 (-0.068, -0.015)	0.085 (0.061, 0.108)	<0.001

HT, hormone therapy; CI, confidence interval.

[a] Restricted to individuals ever used HT.

[b] Derived estimates of turning point and confidence intervals.

[c] Pre-turning point slopes indicated coefficient estimates of regression line before the turning point, while post-turning point slopes indicated regression coefficients afterwards.

[d] Davies' test was performed to test whether there was a change in slope.

© 2024 Liu et al. *JAMA Network Open.*

eTable 11. Segmented Regression Assessing Associations Between Hormone Therapy and Phenotypic Age Discrepancy, Excluding Women With Early Menopause

HT usage [a]	Estimated turning point (95% CI) [b]	Estimated slopes [c]		*P* for differences in slopes [d]
		Pre-turning point (95%CI)	Post-turning point (95%CI)	
Age started HT, y	52.9 (50.0, 55.8)	-0.037 (-0.061, -0.014)	0.033 (-0.026, 0.091)	0.195
HT usage duration, y	10.0 (6.7, 13.3)	-0.018 (-0.035, -0.002)	0.063 (0.001 0.125)	0.017

HT, hormone therapy; CI, confidence interval.

[a] Restricted to individuals ever used HT.

[b] Derived estimates of turning point and confidence intervals.

[c] Pre-turning point slopes indicated coefficient estimates of regression line before the turning point, while post-turning point slopes indicated regression coefficients afterwards.

[d] Davies' test was performed to test whether there was a change in slope.

© 2024 Liu et al. *JAMA Network Open.*

eTable 12. Nonresponse Analysis Comparing Baseline Characteristics of Included and Excluded Postmenopausal Women Participants in the UK Biobank

**Characteristics** [a]	**Excluded N=47 606**	**Included N= 117 763**	***P*** [b]
Chronological age, mean (SD), y	60.3 (5.6)	60.2 (5.4)	0.002
Phenotypic age, mean (SD), y	53.1 (8.2)	52.1 (7.9)	<0.001
White ethnicity	45,198 (94.9)	112,498 (95.5)	<0.001
Higher education	20,309 (42.7)	52,945 (45.0)	<0.001
Annual household income ≥ £31 000	14,843 (31.2)	39,352 (33.4)	<0.001
Employed	44,010 (92.4)	109,583 (93.1)	<0.001
Townsend Deprivation Index below median	22,847 (48.0)	58,824 (50.0)	<0.001
Moderate-to-vigorous physical activity ≥ 150 minutes/week	31,590 (66.4)	84,278 (71.6)	<0.001
Tobacco/nicotine exposure score, mean (SD)	78.4 (30.9)	79.5 (30.3)	<0.001
Chronic kidney disease	1576 (3.3)	3775 (3.2)	0.282
Diabetes	2538 (5.3)	5682 (4.8)	<0.001
Hypertension	27,074 (56.9)	66,446 (56.4)	0.098
Cardiovascular disease	3108 (6.5)	6980 (5.9)	<0.001
Cancer	7065 (14.8)	16,554 (14.1)	<0.001
Bilateral oophorectomy	3140 (6.6)	6065 (5.2)	<0.001
Hysterectomy	6656 (14.0)	12 715 (10.8)	<0.001

[a] Data represented characteristics as mean (SD) or n (%).

[b] P value reported for differences between groups using t test or chi-square test.

© 2024 Liu et al. *JAMA Network Open.*

eFigure 1. Participant Selection Diagram

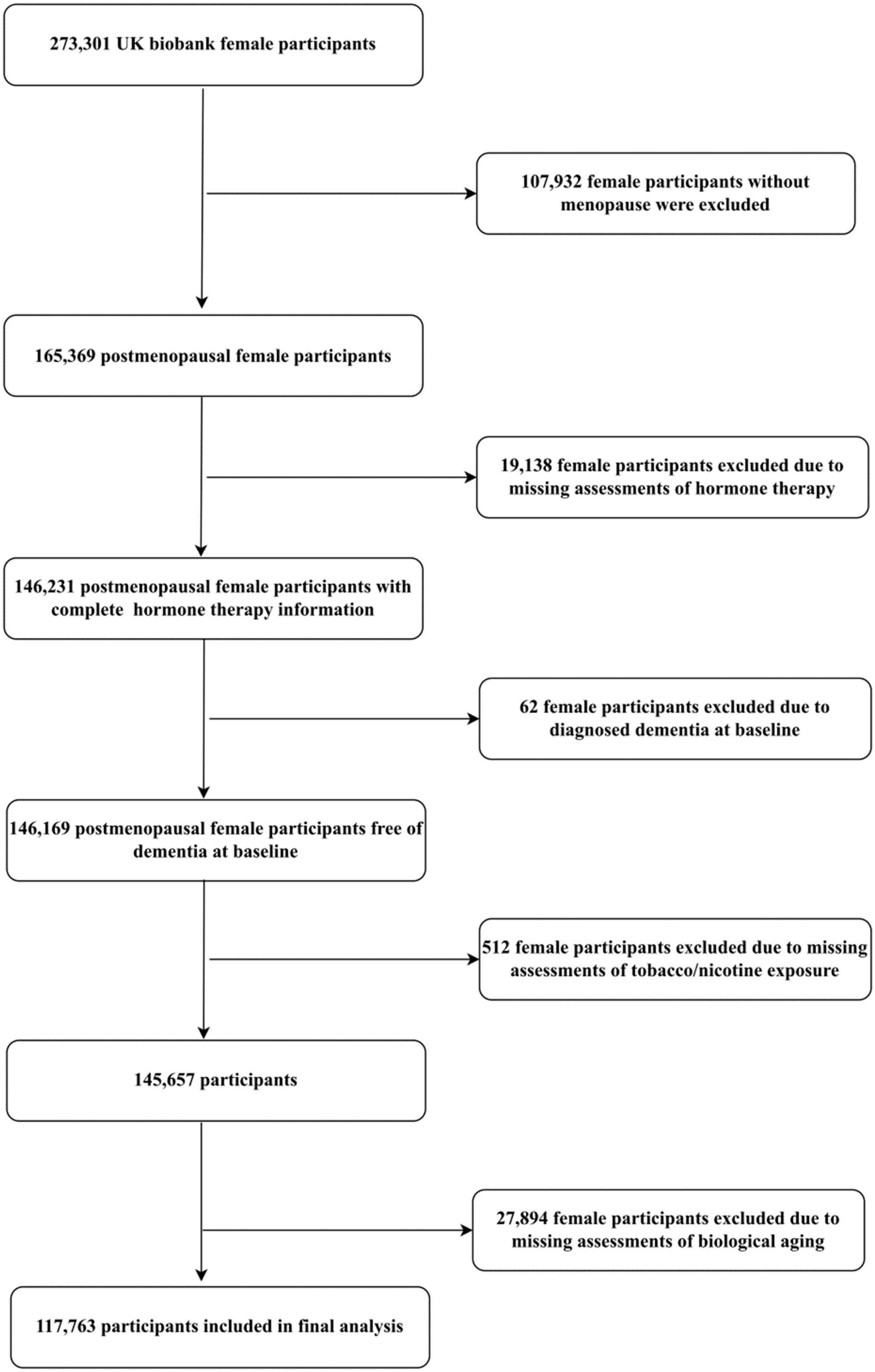

© 2024 Liu et al. *JAMA Network Open.*

eFigure 2. Dose-Response Associations Between Age Started and Years Used Hormone Therapy and Phenotypic Age and Phenotypic Age Discrepancy

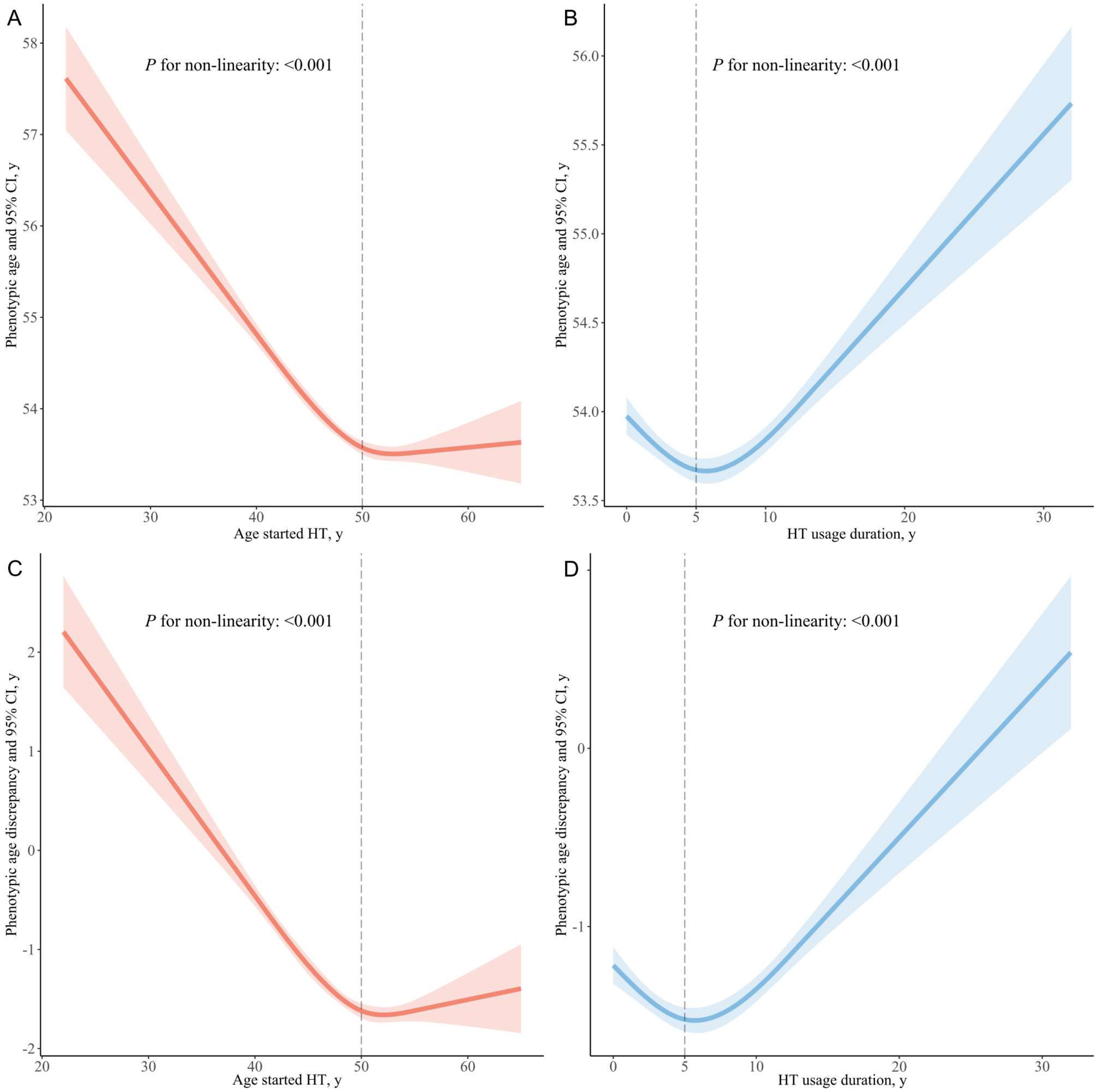

HT, hormone therapy; CI, confidence interval.

Restricted cubic spline models were applied to model each curve, with 3 or 4 knots selected based on model fitting statistics. The analysis was restricted to individuals ever use hormone therapy. Solid lines represent the point estimates of phenotypic age and age discrepancy (y), while shadows represent corresponding 95% CIs. P values for nonlinearity were calculated using the Wald chi-square test.

© 2024 Liu et al. *JAMA Network Open*.

eFigure 3. Segmented Regression Assessing Dose-Response Associations Between Age Started and Years Used Hormone Therapy and Phenotypic Age Discrepancy

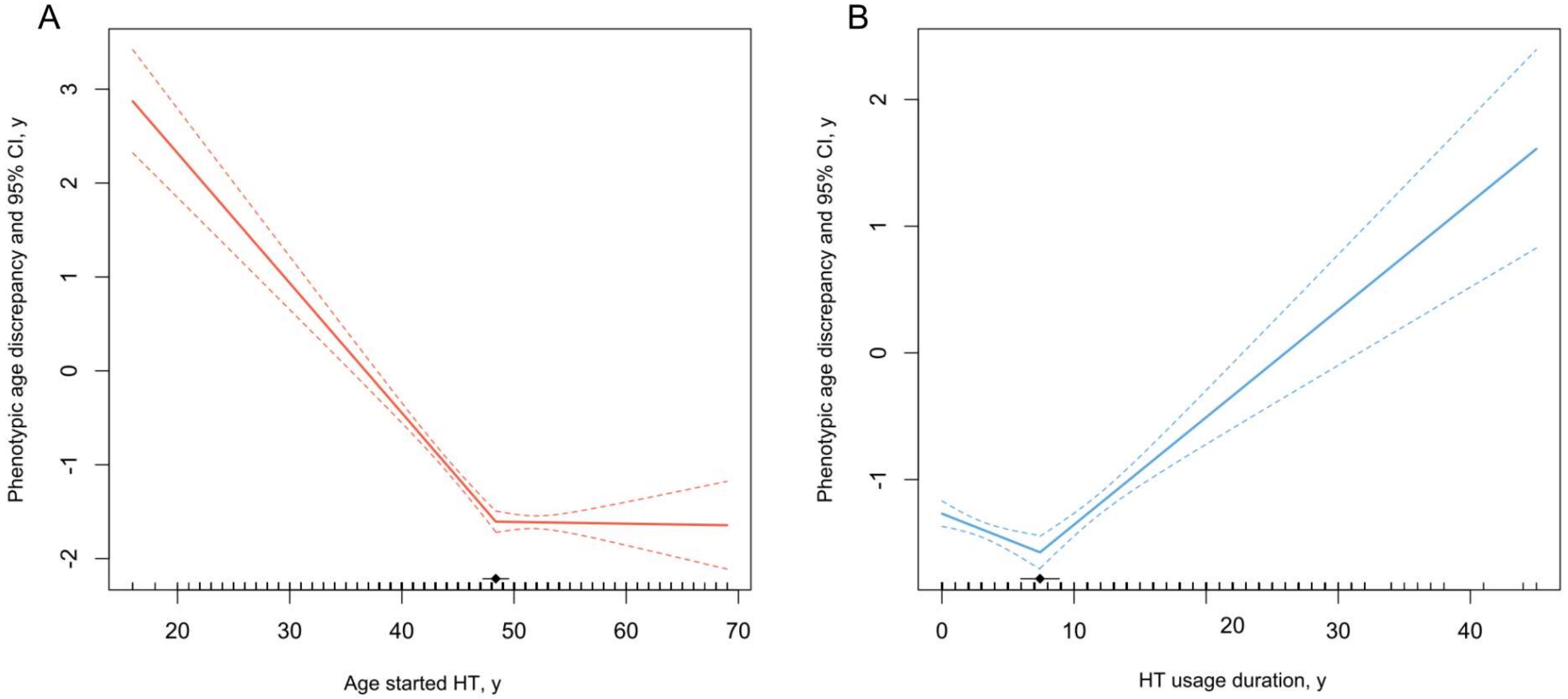

HT, hormone therapy; CI, confidence interval. Solid line represents segmented regression model, while dashed lines indicate 95% CIs. Marks under the line indicate the position of the breakpoint, while density of observations was plotted at the x axis.

© 2024 Liu et al. *JAMA Network Open.*

eFigure 4. Dose-Response Associations Between Phenotypic Age Discrepancy and All-Cause and Cause-Specific Mortality

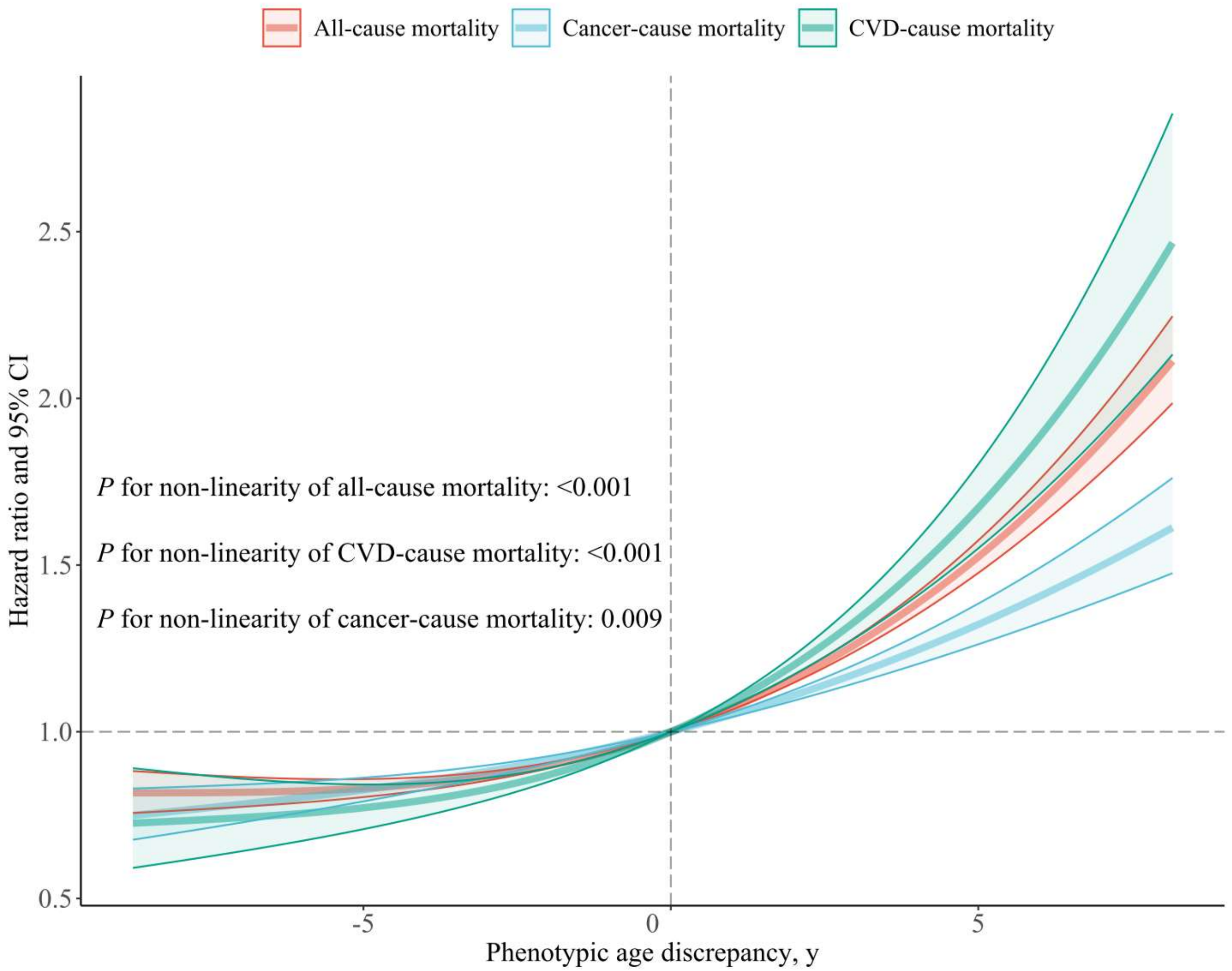

CVD, cardiovascular disease; CI, confidence interval.

Restricted cubic spline models were applied to model each curve, with 3 or 4 knots selected based on model fitting statistics. Solid lines represent the point estimates of hazard ratios of mortality, while shadows represent corresponding 95% CIs. P values for nonlinearity were calculated using the likelihood-ratio chi-square test.

© 2024 Liu et al. *JAMA Network Open*.
