# Supplementary material for: Hormone Therapy and Biological Aging in Postmenopausal Women
Source: JAMA Netw Open. 2024 Aug 29;7(8):e2430839. doi: 10.1001/jamanetworkopen.2024.30839 (PMC11362863; doi:10.1001/jamanetworkopen.2024.30839)
Supplement: Supplement 2. — Data Sharing Statement [file jamanetwopen-e2430839-s002.pdf]

Data Sharing Statement

Liu. Hormone Therapy and Biological Aging in Postmenopausal Women. *JAMA Netw Open*. Published August 29, 2024. doi:10.1001/jamanetworkopen.2024.30839

Data
Data available: No

Additional Information
Explanation for why data not available: The data that support the findings of this study are available from the UK Biobank project site, subject to registration and application process. Further details can be found at https://www.ukbiobank.ac.uk.
